# Insight into the Binding of First- and Second-Generation PET Tracers to 4R and 3R/4R Tau Protofibrils

**DOI:** 10.1021/acschemneuro.3c00437

**Published:** 2023-08-28

**Authors:** Junhao Li, Amit Kumar, Bengt Långström, Agneta Nordberg, Hans Ågren

**Affiliations:** † Department of Physics and Astronomy, Uppsala University, Box 516, SE-751 20 Uppsala, Sweden; ‡ Department of Neurobiology, Care Sciences and Society, Division of Clinical Geriatrics, Center for Alzheimer Research, Neo, 141 84 Stockholm, Sweden; § Department of Chemistry - BMC, Uppsala University, Box 516, SE-751 20 Uppsala, Sweden; ∥ Theme Inflammation and Aging, Karolinska University Hospital, S-141 86 Stockholm, Sweden; ⊥ College of Chemistry and Chemical Engineering, Henan University, Kaifeng, Henan 475004, P. R. China

**Keywords:** 4R tau fibrils, metadynamics, positron emission tomography tracer, free energy surface, Alzheimer disease, molecular dynamics

## Abstract

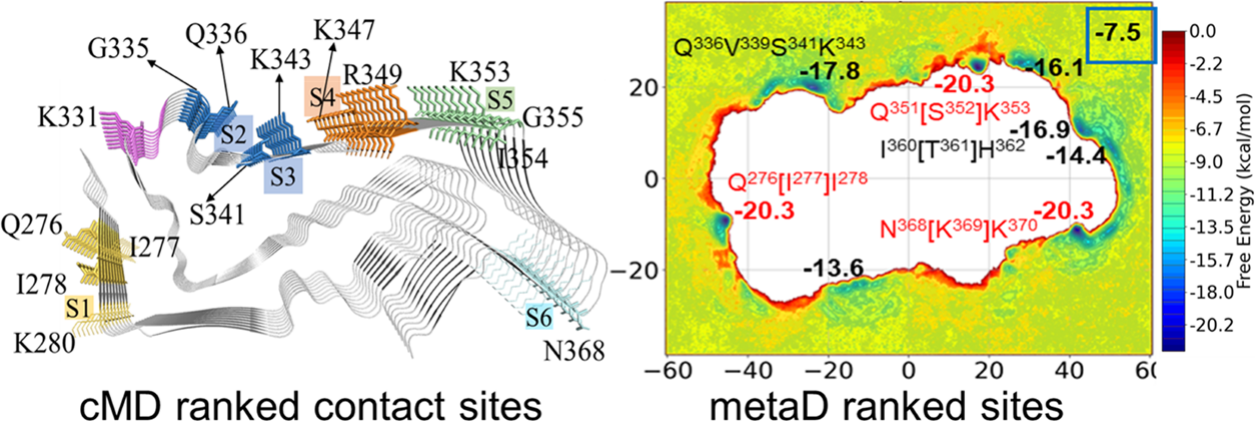

Primary supranuclear palsy (PSP) is a rare neurodegenerative disease that perturbs body movement, eye movement, and walking balance. Similar to Alzheimer’s disease (AD), the abnormal aggregation of tau fibrils in the central neuronal and glial cells is a major hallmark of PSP disease. In this study, we use multiple approaches, including docking, molecular dynamics, and metadynamics simulations, to investigate the binding mechanism of 10 first- and second-generations of PET tracers for PSP tau and compare their binding in cortical basal degeneration (CBD) and AD tauopathies. Structure–activity relationships, binding preferences, the nature of ligand binding in terms of basic intermolecular interactions, the role of polar/charged residues, induced-fit mechanisms, grove closures, and folding patterns for the binding of these tracers in PSP, CBD, and AD tau fibrils are evaluated and discussed in detail in order to build a holistic picture of what is essential for the binding and also to rank the potency of the different tracers. For example, we found that the same tracer shows different binding preferences for the surface sites of tau fibrils that are intrinsically distinct in the folding patterns. Results from the metadynamics simulations predict that PMPBB3 and PBB3 exhibit the strongest binding free energies onto the Q^276^[I^277^]I^278^, Q^351^[S^352^]K^353^, and N^368^[K^369^]K^370^ sites of PSP than the other explored tracers, indicating a solid preference for vdW and cation−π interactions. Our results also reproduced known preferences of tracers, namely, that MK6240 binds better to AD tau than CBD tau and PSP tau and that **CBD2115**, **PI2620**, and **PMPBB3** are 4R tau binders. These findings fill in the well-sought-after knowledge gap in terms of these tracers’ potential binding mechanisms and will be important for the design of highly selective novel PET tracers for tauopathies.

## Introduction

Proteinopathies is a broad umbrella term used to represent neurodegenerative disorders that are characterized by different target proteins and featured by the formation of abnormal protein aggregates in the brain, such as amyloid-β (Aβ), tau, and α-synuclein.^1−5^ Alzheimer’s disease (AD) and Parkinsonian syndromes are the most representative neurodegenerative disorders that are broadly spreading over the world.^6^ Cortical basal degeneration (CBD) and primary supranuclear palsy (PSP) represent atypical forms of Parkinsonian syndromes and are considered as primary tauopathies with an underlying brain aggregation of tau proteins.^7,8^ It has been reported that most of these abnormal protein aggregates are present in the brain much in advance of the appearance of dementia symptoms.^9^ It has therefore become of high relevance to visualize these harmful aggregations of the tau protein in the brains of living patients to detect the disease at an early stage.^2,3,10,11^ However, the research in this area has been hampered by the fact that imaging of tau fibrils is much more complicated than imaging of Aβ amyloids because the tau proteins are embedded inside the neuronal cells, and there are different isoforms of the tau protein with different folding pathological subtypes. Tau fibrils from AD are comprised of mixed 3/4-repeat (3/4R) microtube binding domains, while only 4-repeat tau (4R tau) aggregates are identified in CBD and PSP.^12−16^ Additionally, these fibrils are distributed in different sites/regions in the AD/CBD/PSP brains, and the distributions are also related to the progress of dementia.^17^

Positron emission tomography (PET) has been widely used as a noninvasive imaging tool for neuro disorder diseases.^18^ Compared to other imaging techniques, PET has a higher sensitivity in detecting the γ rays emitted from positron–electron annihilation, where the positrons and electrons originate from the isotope decay of the labeled small tracer molecules and the atoms of the surrounding brain tissue, respectively. The first generation of tau PET tracers including **Flortaucipir** (also named AV1451 or T807),^19,20^**TKH5351**,^21^ and **PBB3**,^22^ show binding both *in vitro* and *in vivo* to both AD (3/4R tau) as well as PSP/CBD (4R tau).^11^ Among the second-generation tau PET tracers, **RO948**(23) and **MK6240**(24) selectively show high binding in AD and no binding in non-AD tauopathies, while **PI2620**(25) and **PMPBB3** (also named **APN1607**)^26^ show high binding in the AD brain tissue, but also in the PSP/CBD brain tissue. The *in vitro* binding data in AD and PSP brain tissues for **PI2620**, **PMPBB3**, and **CBD2115** seem to be comparable with *K*_d_ values in the nM range.^27−31^

*In silico* modeling is a powerful aid in structural-based tracer design.^32^ Several *in silico* binding studies with different tracers have been reported for AD, CBD, and PSP tau protofibrils.^33−37^ Some interior tracer binding sites in the protofibrils (e.g., the e1 site in CBD tau) usually exhibit much stronger binding free energies than the fully exposed surface sites.^35^ However, the interior sites can be difficult to access in a real tissue with many long-range fibrils^36^ because the entrance to these sites of each real fibril may just interact with the body of other fibrils. Our previous simulations for **PI2620** on AD tau protofibrils have proved a high energy barrier for the binding of the tracer into the core site C1.^34^ Interestingly, there are also partially solvent-exposed surface sites for AD tau fibrils due to its special V-shape (groove) folding pattern.^14^ This leads to a configuration of a small molecule binding pocket that is closer to the traditional binding pocket on AD tau fibrils.^28^ Compared to the fully solvent-exposed surface sites on CBD and PSP tau fibrils, the tracer can be less exposed to the solvent environment in these groove sites.^37^ This may explain why most of the first and second generations of tau tracers show less (or no) binding to 4R tau than to AD tau fibrils.

We use a series of computational tools to investigate the 10 selected first and second generations of tau PET tracers with different scaffolds. Extensive docking studies were first performed to initially rank the binding sites throughout all of the surface sites for PSP, CBD, and AD tau protofibrils. The binding of these tracers to the top-ranked sites is presented and discussed. From molecular dynamics (MD) simulations, we observed large movements on some tracers that show weak binding affinities on some specific sites from docking studies. The probability of tracer binding to new sites is also observed. Finally, we performed metadynamics (metaD) simulations to evaluate the binding free energy profiles for 6 tracers on the surface sites of PSP tau protofibrils. We believe that the mechanistic insight at the atomic level, as provided in this *in silico* work, may be used to guide the further development of 4R-specific tracers.

## Results and Discussion

### Determination of Surface Binding Sites for Tracers from Docking Studies

**Figure 1:**
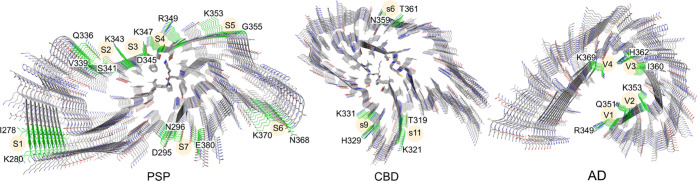
Structures of and ranked surface binding sites for the 9-chain PSP (numbering after “S”), CBD (numbering after “s”), and AD (numbering after “V”) tau protofibrils.

A large amount of compounds with different scaffolds have been reported to be potential PET tracers for the imaging of tau fibrils.^11^ However, tracers exhibiting high selective binding toward 4R tau fibrils have not yet been reported, especially the PSP tau fibril. To start, we selected from literature data 10 compounds with various scaffolds, including nitrogen-embedded polycyclic aromatic rings (**Flortaucipir**, **RO948**, **PI2620**, and **GTP1**), pyridine-indole (**CBD2115**), buta-1,3-dienyl benzothiazole (**PBB3**, **PMPBB3**), naphthyridine (**JNJ311**), pyrrolo-pyridine (**MK6240**), and pyridine-quinoline (**THK5351**), for the docking studies on the surface binding sites (Figure 1) on the 9-chain CBD, AD, and PSP tau protofibrils.

By comparing the Cryo-EM structures of PSP,^15^ CBD,^38^ and AD tau protofibrils,^28^ the volumes of the interior cavities were calculated and follow the order of PSP > CBD > AD (Figure S1). Künze et al. evaluated the associated binding constant of **PI2620** to the cavity sites of CBD and PSP tau protofibril by Brown dynamics simulations,^37^ indicating a very slow intercalation of the tracer to the interior cavity sites due to the low *k*_assoc_ values. In this study, we focus on comparing the binding on the surface sites of different tracers on PSP, CBD, and AD tau protofibrils. Based on the results from molecular docking and a previous study on CBD and AD protofibrils,^35^ we selected out 7 and 3 surface sites for PSP and CBD tau, respectively, and 4 concave sites located in the groove of the AD tau protofibril for MD simulations.

**Figure 2:**
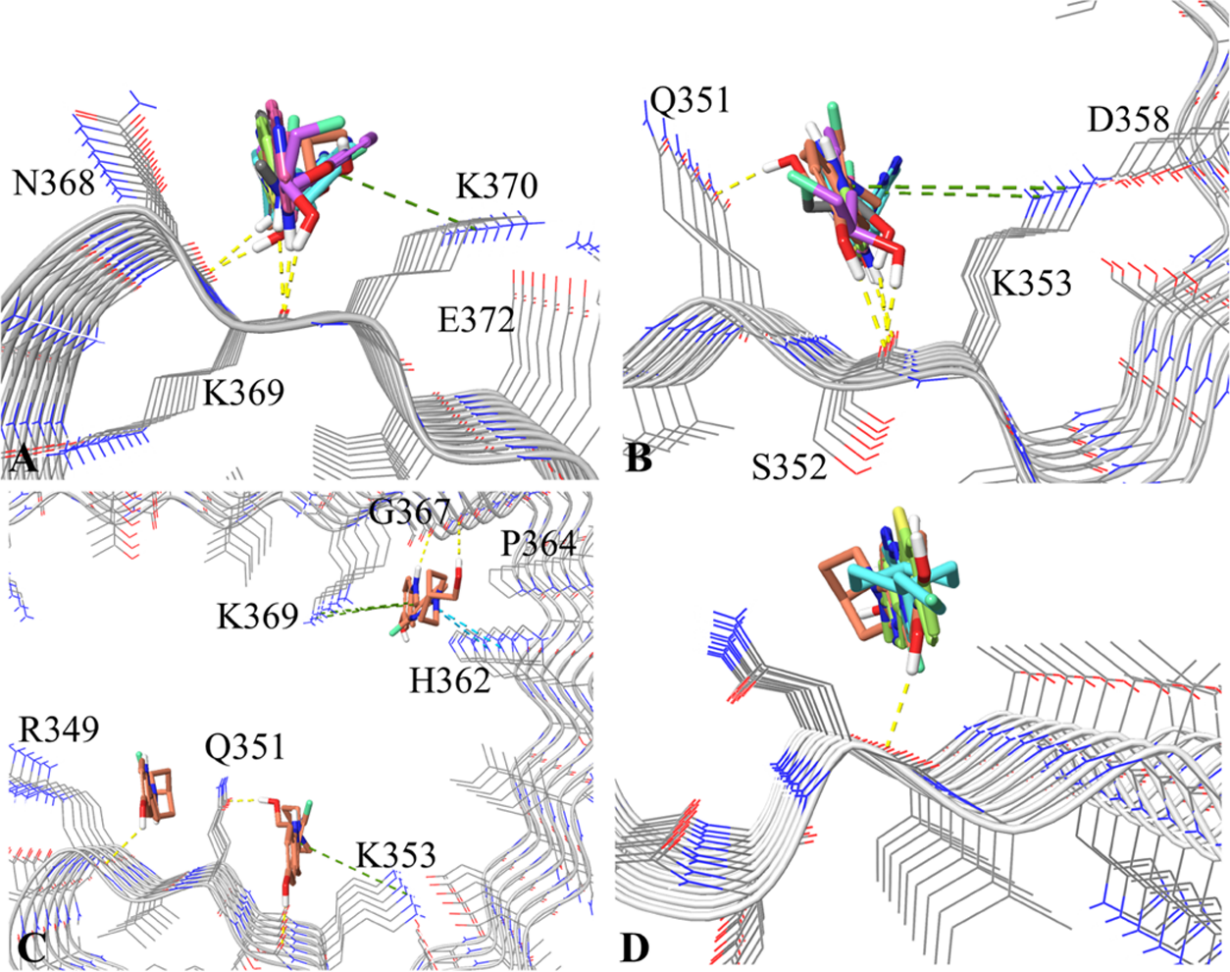
(A) Superimposed binding modes of **PI2620**, **GTP1**, **CBD2115**, **PBB3**, **PMPBB3**, and **THK5351** at the S6 site of PSP tau. (B) Superimposed binding modes of **PI2620**, **GTP1**, **CBD2115**, **PBB3**, **PMPBB3**, and **THK5351** at the V2 site of AD tau. (C) Superimposed binding modes of **CBD2115** at the V1, V2, and V4 sites of AD tau. (D). Superimposed binding modes of **CBD2115**, **PBB3**, and **GTP1** on the s6 site of CBD tau. The hydrogen bonds, cation-π, and π–π interactions are depicted as yellow, green, and cyan dash lines, respectively.

From the docking calculations, we found that site S6 (Figure 1) exhibits the highest binding affinities for the binding of 6 selected tracers (**PI2620**, **CBD2115**, **PBB3**, **PMPBB3**, and **THK5351**) on the surface of the PSP tau protofibrils. Site S6 is constituted by the side-chain/backbone atoms of N368 and K370 and the backbone atoms of K369 (denoted as N^368^[K^369^]K^370^; the residue in the bracket has very few side-chain interactions with the tracer, and this will used throughout the text). The backbone of K369 and N368 can serve as hydrogen bond acceptors to contact the hydrogen bond donors in the tracers (Figure 2A). Due to the adjacent E372, the distance between the positive amine group and aromatic rings of the tracers is somehow too far for suitable cation−π interactions. It is notable that the concave sites V2 of AD tau protofibrils also have a similar configuration of residues (Q^351^[S^352^]K^353^, Figure 2B). The docking scores between the V2 and S6 sites are generally similar for the tracers **THK5351**, **PBB3**, **GTP1**, **PI2620**, **CBD2115**, and **PMPBB3**.

We observed that **CBD2115** and **PMPBB3** can show high binding affinities in that the docking score could reach −7 to −8 kcal/mol for some sites in both AD and PSP tau fibrils. The docking scores at AD tau are better than those on PSP tau fibrils. Both of **CBD2115** and **PMPBB3** show moderate binding affinities (−6.0 to −6.7 kcal/mol) to the surface sites of CBD tau protofibrils. **CBD2115** exhibits very high binding affinity at the AD tau site V4 with H^362^P^364^[G^367^N^368^]L^369^ (Figure 2C). **PBB3** also shows many binding sites with docking scores in the range of −6 to −7 kcal/mol on the surface of PSP (S1, S4, and S6) and CBD (s6, s9, and s11) tau with comparable docking scores to the AD tau fibrils (Table 1).

**Table 1:**
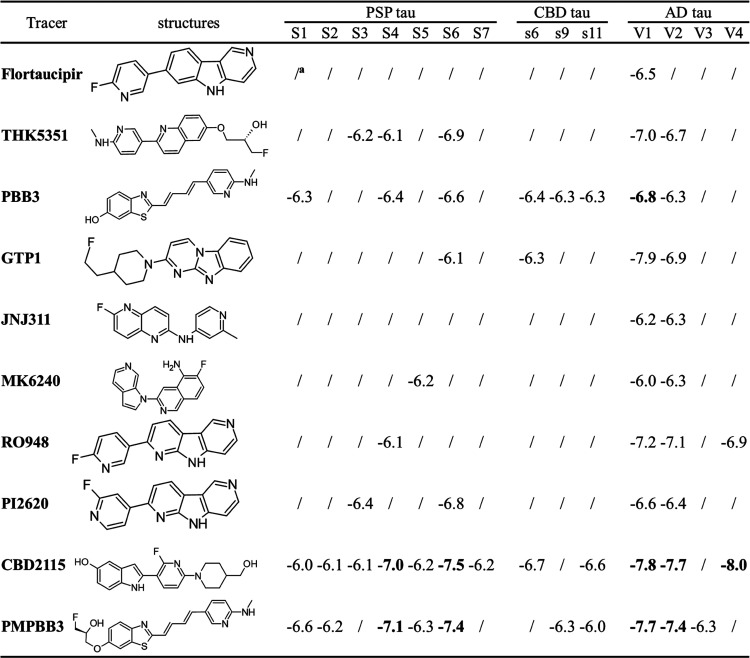
Docking Scores of 10 Selected Tracers on Different Surface Sites of PSP, CBD, and AD tau Protofibrils

a Low binding, docking score worse than −6.0 kcal/mol.

The s6 site (N^359^[I^360^]T^361^) of CBD tau ranked from blind docking is a unique site without a side-chain of basic residues. For the site-specific CBD docking studies, s6 exhibits higher binding affinities for **GTP1**, **CBD2115**, and **PBB3** than the s9 and s11 sites (Figure 2D). Through docking studies, we identified the initial binding modes of the 10 selected tracers to the surface sites of PSP, CBD, and AD tau fibrils. The AD tau sites are located in the concave groove due to their folding pattern, which provides more contact to the tracers. The site S6 in the PSP tau protofibril is similar to the V2 site in AD tau and also fits well with the binding of most of the tracers.

The docking-derived 140 complexes were then subjected to MD simulations. Here, we also performed more docking studies using Autodock4^39^ with a similar grid box setting in our previous study.^34^ The internal cavity sites were much better ranked than the surface sites that are generally scored in the range of −5.0 to −6.0 kcal/mol and partially overlapped with the Glide docking results (Figure S2).

### Mobility of Tracer Binding on the Surface Sites Evaluated by Molecular Dynamics Simulations

From the docking-derived initial binding modes of these tracers, the compensation of the solvent effect and protein flexibility was introduced by performing a 100-ns MD simulation for each compound at each site of the three protofibrils. The time scale of 100-ns is often insufficient to observe the unbinding of small molecules from an enclosed binding site; however, the folding pattern of pathological tau proteins exposes the ligand binding sites to the solvent, and dissociation can, therefore, be observed due to a smaller number of contacts between the ligand and its surface binding sites.^37^ For example, the binding and unbinding of 60 bTVBT4 molecules onto a full periodic model of the AD tau fibril could be easily observed in the simulations performed by Todarwal et al.^36^

We inspected all of the trajectories to check if the tracers were stable in the docking-derived binding sites (Table 2). For the PSP protofibril, sites S1 and S7 seem not to be suitable for the binding of all tracers, while site S4 can enclose all of the tracers. Site s6 on the CBD protofibril is unstable for all of the tracers, in which all of the tracers shifted to neighbor regions. The shift may be induced by the flat shapes of the nearby residues of site s6 (Figure 1). For the AD protofibrils, V3, the site neighboring the V4 site, is the most unstable site for tracer binding, whereas V4 is a small groove-shaped site gated by H362 and N368 (Figure 1). We can see at a gross level that the binding modes of **PBB3**, **RO948**, **PI2620**, **CBD2115**, and **PMPBB3** are relatively stable in most of the docking identified binding sites.

**Table 2:**
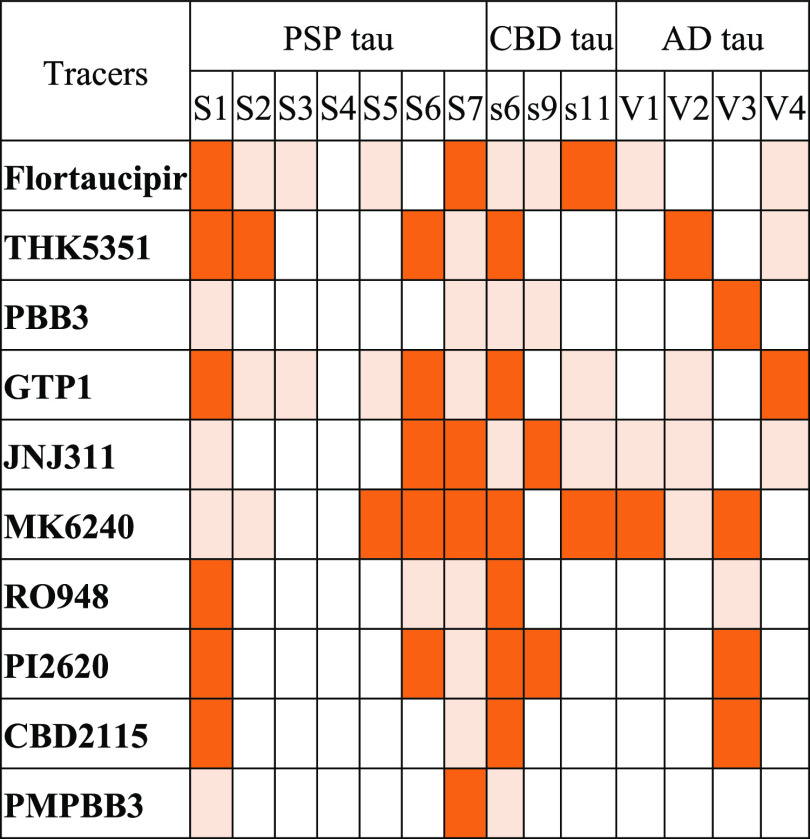
Overview of the Tracer Mobilities during MD Simulations^a^,^b^,^c^

a Blank: The tracer remained stable in the initial binding pocket.

b Light orange: The tracer left the initial site after 50-ns.

c Orange: The tracer left the initial site quickly within 50-ns.

**Figure 3:**
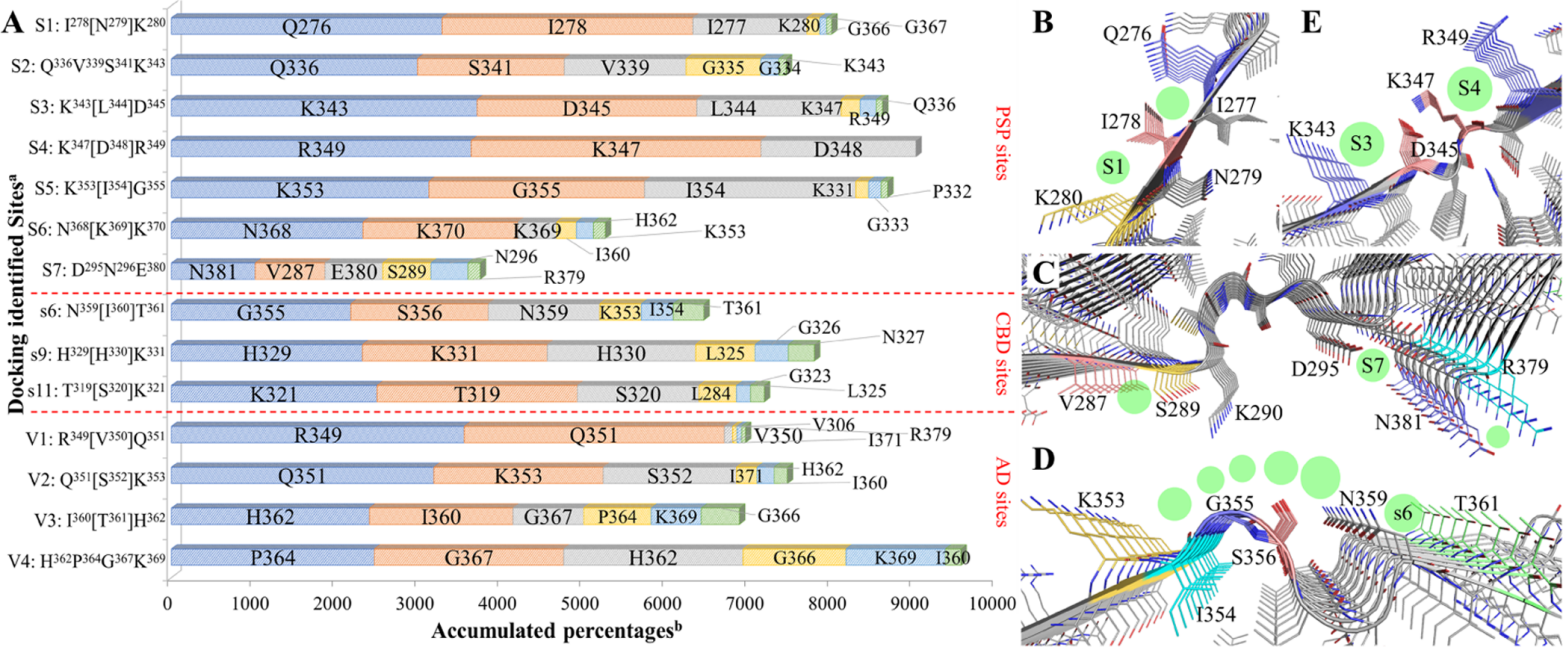
Overview of the surface residues that come into contact with tracers during the 100-ns MD simulations. (A) Statistical data of the contacts. A threshold distance of 4.0 Å between the non-hydrogen atoms of the protein residues and tracers was used to count the contacts. The top 6 most contacting residues are depicted. ^a^Configuration of each docking identified site is given for comparison. ^b^Percentages are accumulated from the MD simulations of each tracer over all of the protofibril chains. (B), (C), and (D) Configurations of new sites (green spheres) in the MD simulations of docked sites (green spheres with labels) S1, S7, and s6, respectively. (E) Comparison of the sites S4 and S3 on the PSP protofibril.

**Figure 4:**
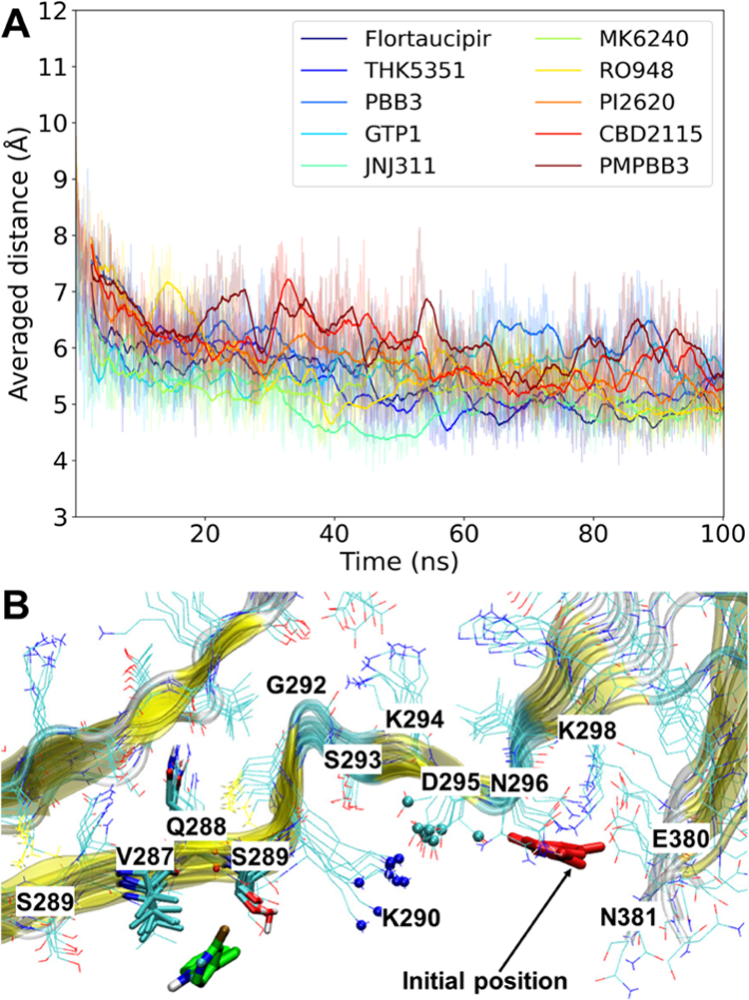
(A) Time course of the averaged distance of the “NZ” atoms of K290 and the “CG” atoms of D295 for all of the 9 chains during the 10 MD simulations with different tracers at site S7. (B) Representative snapshot of **PI2620** falling into site V^287^[Q^288^]S^289^ from the simulation starting from PSP site S7. The “NZ” atoms of K290 and “CG” atoms of D295 are depicted in blue and cyan spheres, respectively. **PI2620** and the residues within 4.0 Å of **PI2620** are rendered as cyan and green sticks, respectively.

In many cases, though the unbinding to the initial docked sites is observed, the tracer can either stay in the solvent or bind to the other sites. So, we performed contact analysis for all of the trajectories by counting the snapshots with the non-hydrogen atoms of the tracer and protein being closer than 4 Å (Figure 3A). In the MD simulations for these sites, tracers shifted the major contacts with neighboring Q^276^[I^277^]I^278^, V^287^[Q^288^]S^289^, and G^355^S^356^K^353^[I^354^] for the docked S1, S7, and s6 sites, respectively (Figure 3B–D). The side-chain atoms of I278 became the wall between site S1 and the new site (Figure 3B). Site S7 on the PSP protofibril exhibits the least capacity for enclosing the tracers (Figure 3A). The major tracer binding site starting from S7 shifted to the neighboring R^379^N^381^ and a middle-distance site V^287^[Q^288^]S^289^, which is separated by a small groove between D295 and K290 (Figure 3C). However, this groove is found to close up due to the formation of the D295-K290 salt bridges, laying down the conformation of K290’s side-chain atoms (Figure 4). Such dynamical structural bias explains why V^287^[Q^288^]S^289^ becomes a competitive binding site (845% accumulated portion) to S7 (700%, Figure 3A) because the space barrier between V^287^[Q^288^]S^289^ and S7 was flattened. Site S7, located at β13, was also found by Künze et al.^37^ to be unstable for the binding of **PI2620** with a positive binding free energy. For the CBD protofibril, site s6 was found to be extended to more neighboring residues, K^353^[I^354^]G^355^S^356^, indicating an orientation of tracers that is perpendicular to the axis of the protofibril (Figures 3D and S3). In contrast to S1, S7, and S6, site S4 (K^347^[D^348^]R^349^) is found to fit all of the tracers with the accumulated portion of more than 3500% and without other contacts (Figure 3A,E). Moreover, from the row of site S3 (Figure 3A), we can see that ∼200% of accumulated contacts shifted to the neighboring S4 site. Interestingly, S4 is mainly configurated by the side-chain atoms of two basic residues, K347 and R349, with the lysine forming a salt bridge with D345. This site is also validated by the free energy surfaces (FES) obtained by Künze et al.,^37^ which indicates a deep local minimum for the binding of **PI2620**. The CBD protofibril also exhibits a similar surface site s9 that is constituted by two basic residues and shows good stability for tracer binding. For the AD tau protofibril, the concave sites are the most identified sites for the PET tracer binding from experimental research.^28,40^ The V4 site has two basic residues on the two sides; however, H362 and K269 are separated by 7 residues. Looking into the sites ranked between the top 3 and top 6 from the simulations starting from V3 and V4, we can identify a certain amount of exchange of tracer binding between the two sites (Figure 3A). The structural and dynamical features of these sites will also play different roles in the binding profiles of these tracers to 4R- and 3R/4R tau fibrils.

**Figure 5:**
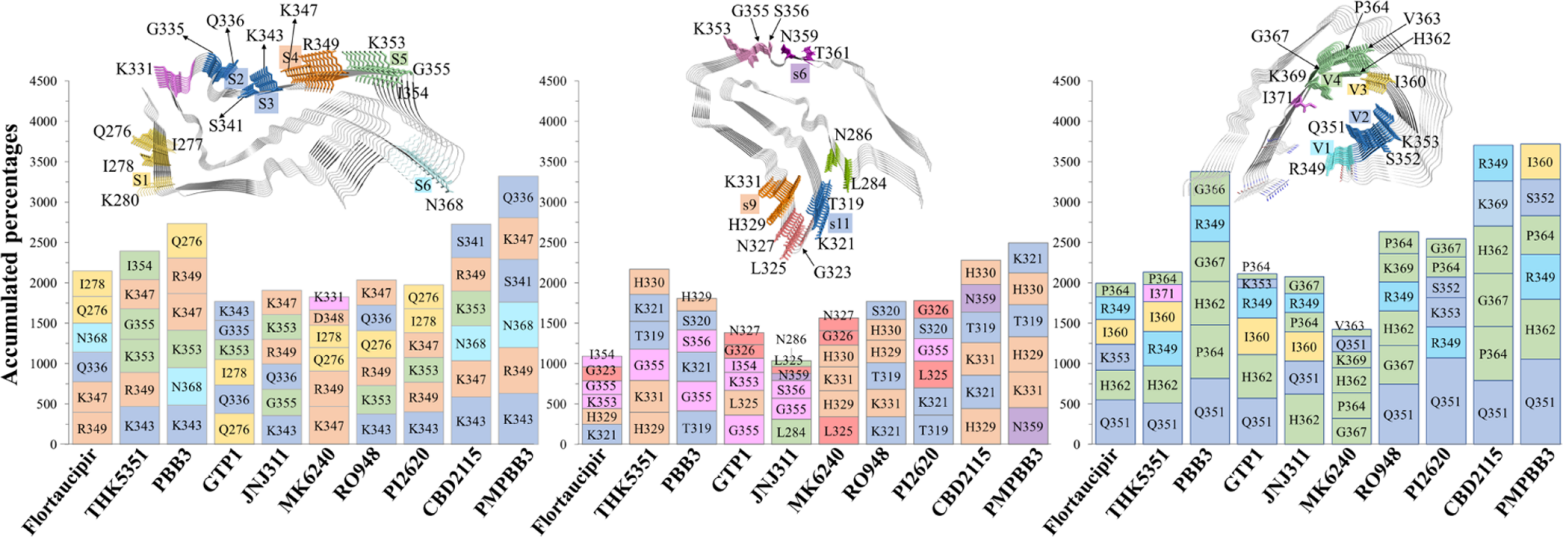
Top 6 most contacted residues for each tracer on PSP (left), CBD (middle), and AD (right) protofibrils throughout 140 (10 tracers * 14 initial sites) 100-ns MD simulations.

We further summarize the overall contacts for ranking the top 6 most contacting residues for each tracer (Figure 5). Although the basic residues are included in the initial sites, the binding pattern of tracers on these protofibrils shows different preference to the basic residue sites, in which PSP shows the highest portion (K343 from site S2 or S3 and R349 from site S4 for all of the tracers except **GTP1**), followed by the CBD (K321 from site s11, H329 from site s6, and R349 from a new site for **Flortaucipir**, **THK5351**, **RO948**, and **CBD2115**) and AD protofibrils (H362 for **JNJ311**). Interestingly, all of the four “V” sites of AD protofibrils contain at least one basic residue (Figure 1). In the V1 and V2 sites, the distances of R349-Q351 and K353-Q351 pairs are slightly enlarged after MD simulations (Figure S4A), which may be caused by the repulsive interactions between R349 and K375 on the other side of the groove. Such repulsive interaction also slightly enlarges the mouth of this groove (Figure S4B), providing a reasonable explanation for the better preference for contacting the basic residues of the PSP and CBD protofibrils. The accumulated portion of contacts for each top 6 ranked residue on CBD seems to be lower than those on the PSP and AD protofibrils, indicating a larger mobility of tracers on the CBD protofibril. For the binding of PI2620, the top 6 most contacting residues are involved in 5 sites (Q^276^[I^278^]K^280^ near S1, S2, S3, S4, and S5) and 3 sites (s11, extended s6, and L^325^[G^326^], Figure 5) for the PSP and CBD protofibrils.

**Figure 6:**
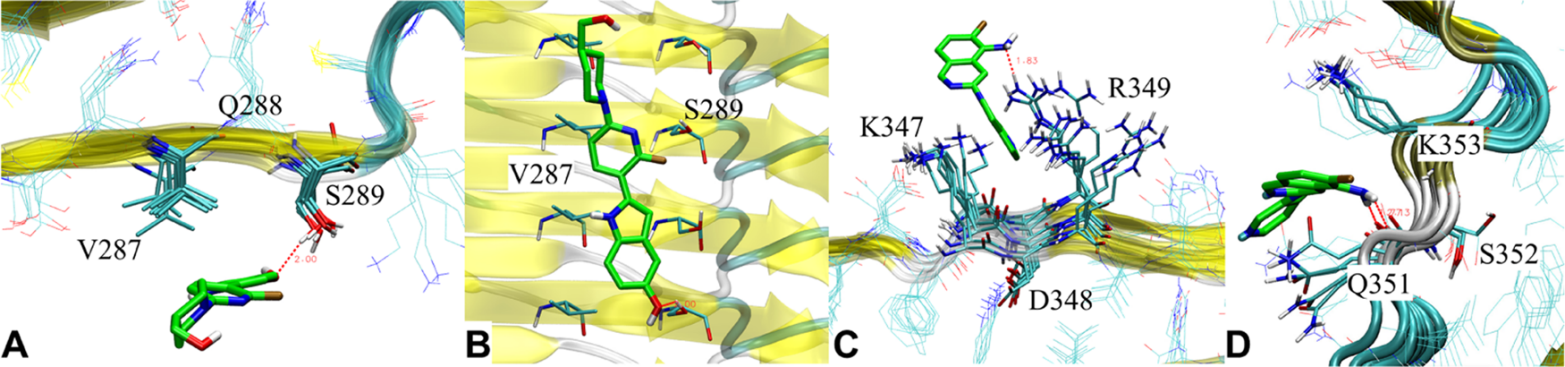
Examples of binding modes. (A) and (B) Representative snapshots of **CBD2115** on the V^287^[Q^288^]S^289^ site of PSP protofibril from the front and bottom view, respectively. (C) Vertical binding mode of **MK6240** on the S4 site of the PSP protofibril (front view). (D) Parallel binding mode of **MK6240** in the V2 site of the AD protofibril.

The protein–tracer contact information has proven that when a tracer leaves the docked surface sites (within or after 50-ns), the tracer could shortly remain at other surface sites that are poorly ranked by the docking. For example, in the last 20-ns simulation of **CBD2115** starting from PSP site S1, the tracer is found to stay in a site V^287^[N^288^]S^289^ (Figure 6A,B) with the accumulated portion of contact of 210% (Figure 3A), which is a competitive site in the simulations starting from site S7. Hydrogen bonding between **CBD2115** and V^287^[N^288^]S^289^ can be observed. The simulations for **PBB3**, **CBD2115**, **PI2620**, and **PMPBB3** are found to be relatively stable in most of the PSP surface sites (Table 2), while three of them are second-generation tau tracers. For the experimentally characterized 3R/4R tau binder **MK6240**, although it also exhibits a strong preference for contact with K347 and R349 on the PSP protofibril (Figure 4), we find that its orientation is actually vertical to the axis of the fibril, exposing the large benzopyridine moiety to the solvent environment (Figure 6C). The binding mode of **MK6240** on PSP is, therefore, different in all of the sites on the AD protofibril (Figure 6D), which is also observed in a previous small section of autoradiography.^31^

The time length of simulations may impact the distribution of the tracer most contacted sites. Additionally, we performed 70 (starting from sites S1-S7 for each of the 10 tracers) 500-ns MD simulations for the tracers on PSP protofibril with new seeds generating initial velocities. Compared to the simulations with a length of 100-ns, the top-1 and top-2 most contacted residues changes for all of the systems, but they mostly belong to sites S2 or S3 for **THK5351**, **PBB3**, **JNJ311**, **PI2620**, **CBD2115**, and **PMPBB3** (Figure S5). For the top 6 most contacted sites, the scope of contacted residues is similar to the distribution derived from the simulation length of 100-ns. We believe that the simulation length of 100-ns starting from multiple sites is generally sufficient for studying the mobility of tracers.

In the docking top-ranked CBD surface site without basic residues, s6, the simulations were unstable for all of the tracers that moved to the neighboring sites K^353^[I^354^]G^355^S^356^ or T^361^[H^362^]V^363^ (Figure 3D). Only **CBD2115** moved to T^361^[H^362^]V^363^, while the other 9 tracers fell into K^353^[I^354^]G^355^S^356^. For site s9, H^329^[H^330^]K^331^, **RO948**, **CBD2115**, **PBB3**, **PMPBB3**, and **MK6240** were found to be stable. Since **MK6240** is known as a 3R/4R tau binder (more than 500-fold selectivity over 4R tau in *K*_D_),^24,41^ site s9 may not be considered as a good site to distinguish the binding of different tau tracers for CBD protofibrils. Site s11 is close to the concave groove of CBD tau, which folds more tightly than that in AD tau. Simulations starting from this site were stable for the binding of **Flortaucipir**, **PI2620**, **CBD2115**, **PBB3**, **PMPBB3**, and **JNJ311**. From the simulation results, the bias of selecting only a few sites (especially for the CBD protofibril) was compensated to some extent because new surface sites were observed during the MD simulations.

A very recent paper^40^ has shown a novel and interesting binding mode that multiple **GTP1** molecules can be stacked along the axis of the fibril in the groove site of the AD tau fibril with a very high concentration of tracer, indicating that ligand cooperativity can compensate for the instability of these half (the V2 and V3 sites in the AD protofibril) solvent-exposed binding sites. This also implies a further consideration of the possibility of ligand cooperativity in the fully solvent-exposed surface sites for the 4R tau fibrils.

### Free Energy Profiles of Tracer Binding Derived from Metadynamics Simulations

A typical feature of the surface sites for the pathological tau fibril is that they usually contain only 2 to 4 residues that are repeated 4 to 6 times on the axis of the protofibril.^33,34,42^ Through the MD simulations, the dynamics of both tracers and protofibrils can be well captured, such as the new ionic interactions within the protofibril in PSP and the unbinding and rebinding of tracers. However, a qualitative comparison of binding modes of tracers on tau protofibrils often needs further evaluation, such as that given by binding free energy calculations.

To gain energy insight into the binding of these tracers to the surface sites of PSP protofibrils, we further performed well-tempered metadynamics simulations for the tracers of **CBD2115**, **PI2620**, **Flortaucipir**, **PMPBB3**, **PBB3**, and **MK6240**. Reweighting the biased potential obtained from the metadynamics simulations to recover the full FES of the rare events has shown great advantages over the end-point free energy calculations implemented with implicit solvent models.^43−45^ Here, by using the *X* and *Y* coordinates of the center of the tracer as collective valuables (CVs) for the bias potential to act on, the conformational sampling of the tracer is enhanced, and thereby, one can evaluate the favorable sites and strength for the binding of different tracers from the FESs.

The setting of CVs allows the interactions between the tracer and solvent molecules and ions with the strength of −6 to −9 kcal/mol, the averaged energies can be treated as the solvation free energies for each tracer (Figure 4), which should be canceled out for measuring the real binding free energy of the tracer on a specific surface site. There are many strong hydrogen bonds and salt bridges between the folded chains; therefore, the ligand will not crash the inherent structure of the tau fibrils, which leaves a blank area (nonbinding area) on the FES shapes exactly the same as the protofibril viewed from the *z*-axis. It is possible to adopt other sets of CVs to enable the tracer to enter the interior cavities during metadynamics simulations; previous studies have indicated that the entrance of tracers into the interior cavities of the tau protein along other CVs requires relatively high energy barriers.^34,37^ Hence, compared to the conventional MD simulations for which the dissociation on a particular site takes place occasionally, setting CVs along the *X* and *Y* dimensions of the ligand centroid allows us to sample more thoroughly throughout all of the surface residues without crashing the structure of the protofibril and to randomly place the tracers at the beginning.

**Figure 7:**
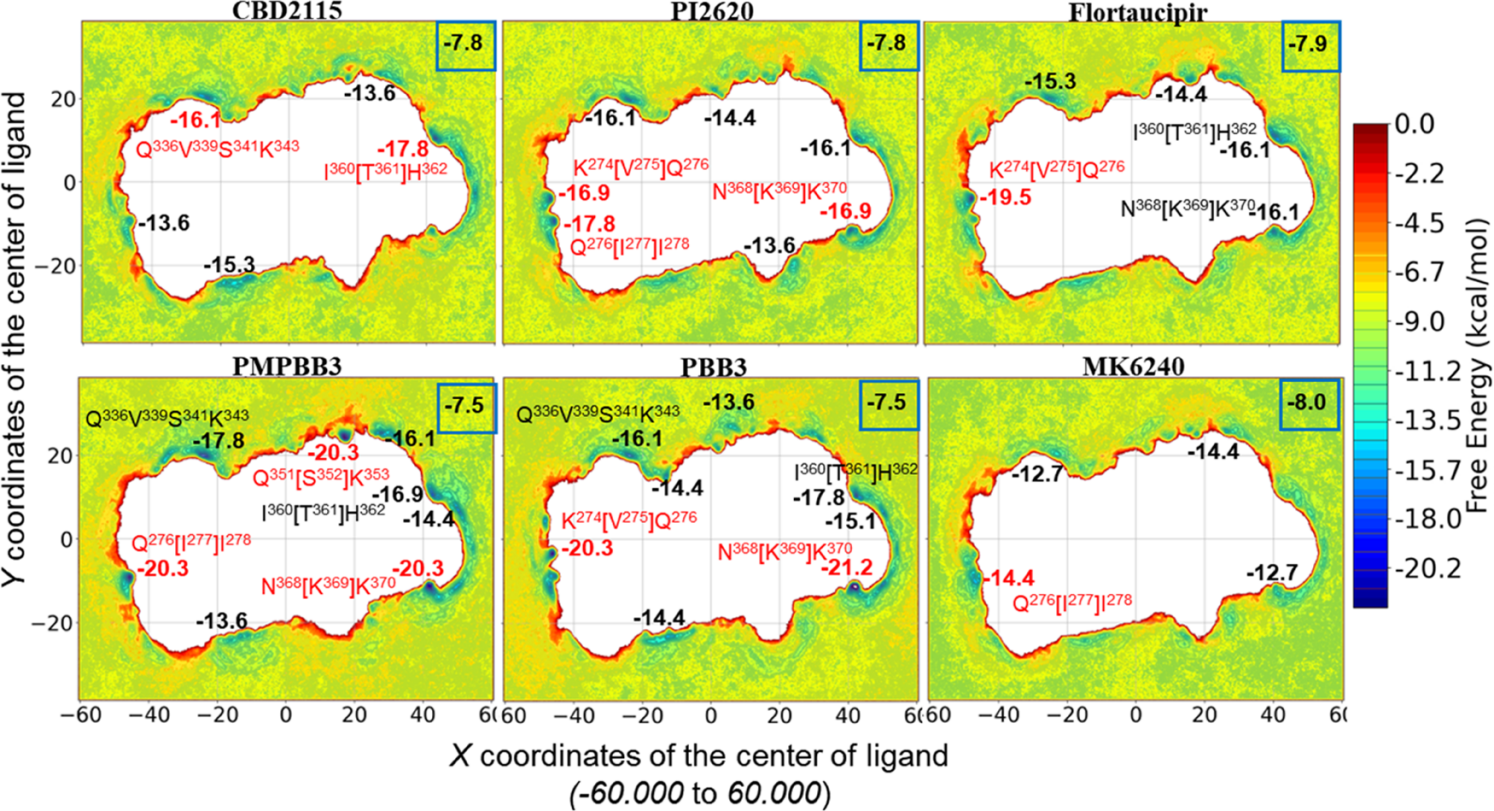
Binding sites and free energy profiles from the 4.5-μs well-tempered metadynamics simulations for 6 selected tracers in PSP protofibrils. CV1: the *X* coordinates of the ligand centroid; CV2: the *Y* coordinates of the ligand centroid. The averaged free energy of the tracer in the solvent and the lowest free energy of some local minima are given in bold numbers with and without a blue frame box, respectively. The free energies of some top-ranked local minima are colored in red, with the label of their binding site configuration marked also in red. The orientation of the PSP protofibril is identical to the one depicted in Figure 1.

From the distributions of the local minima of the simulated systems, all six tracers are not found to bind to the small groove near docking site S7. This agrees with the MD simulations that the ionic interactions between K290 and D295 close the groove formed by residues K290 to D295 (Figure 4). This site was found to be a local minimum for the binding of **PI2620** from the metadynamics performed by Künze et al. (site 7).^37^ As discussed above, in our settings of CVs, the dynamics of protein are not greatly perturbated by the bias potential. **CBD2115**, **PI2620**, **Flortaucipir**, and **PMPBB3** are the know tracers that can bind both 3R/4R and 4R tau fibrils,^25−27,31,46^ while **MK6240** shows few bindings to the 4R tau fibrils. At an overall level, the well depth and numbers of local minima on these FESs also indicate the better PSP binding preference for **PMPBB3**, **CBD2115**, **Flortaucipir**, and **PI2620** than **MK6240** (Figure 7). **CBD2115** exhibits the highest binding affinity of −10.0 kcal/mol (with the cancelation of solvent free energy) to the site I^360^[T^361^]H^362^, followed by a local minimum (−16.1 kcal/mol in Figure 7) at the site Q^336^V^339^S^341^K^343^, which is also the site S2 identified by the docking study. Despite the contribution of more possibility for hydrogen bonding interactions from the hydroxyl group on the indole and piperidine rings, we find that the cation−π interaction or hydrogen bonding is not the leading force for binding compared to **PI2620**. This is, maybe, caused by the piperidine moiety in **CBD2115** that affects the orientations of both hydroxyl groups and the indole and pyridine moieties. In our previous metadynamics simulation for **CBD2115** on the CBD tau protofibril, a similar effect of **CBD2115**’s piperidine ring was observed.^35^ Two neighboring local minima (Q^276^[I^277^]I^278^ and K^274^[V^275^]Q^276^) near the docking identified site S1 show strong preference for the binding for **PI2620**, **PMPBB3**, and **Flortaucipir**. For **PI2620**, both sites can fit the ligand with a slightly higher preference on Q^276^[I^277^]I^278^ (−17.8 kcal/mol). For **Flortaucipir**, the binding is much more, preferring the K^274^[V^275^]Q^276^ site with deeper minima (−19.5 kcal/mol). We speculate that the more linear scaffold of **Flortaucipir** can have better cation-π interactions than **PI2620**. However, on an overall level, the number of deep local minima (<−13.0 kcal/mol) for **PI2620** (7) is larger than for **Flortaucipir** (5), which means more possibility for **PI2620** binding. It may compensate for the overall binding affinity for **PI2620** measure at the tissue level for the real PSP tau fibrils.

The binding of **PMPBB3** and **PBB3** shows three and two highly strong binding sites (<−20.0 kcal/mol) on the FESs, respectively. None of these sites overlaps with the strongest binding site identified for **CBD2115** (I^360^[T^361^]H^362^), which clearly reveals the distinct binding preferences of **PMPBB3** and **CBD2115** and may support the results from a current displacement study by Graham et al.^47^ Our previous metadynamics simulations for CBD and AD tau protofibrils also indicated very high binding for **PMPBB3**.^35^ Recently, Qi et al. identified that **PMPBB3** has the top rank of hydrophobic contacts against other tracers to the 3R/4R chronic traumatic encephalopathy (CTE) tau protofibrils.^48^ These results indicated that the combination of buta-1,3-dienyl and benzothiazole in the scaffold of **PMPBB3** and **PBB3** is a good strategy for binding to the surface sites on both 3R/4R and 4R tau fibrils. The use of 1,3-butadiene to connect the benzothiazole and pyridine rings leads to a longer linear scaffold compared to **CBD2115**, which is believed to be beneficial for contacting with more fibril chains. However, the 1,3-butadiene moiety is also photon-sensitive, which can lead to undesired photoisomerization. Further investigations and improvements for the potential and selective 4R tau PET tracers are still ongoing.

## Conclusions

Motivated by the great need to develop biomarkers for early diagnosis of neurodegenerative diseases and by the outstanding possibilities offered by the rapid development of in silico hard- and software, in this work, we have carried out a computational investigation of first- and second-generation PET tracers for 4R tau fibrils based on fundamental atomic principles. We illustrated the binding of 10 first- and second-generations of PET tracers for PSP tau protofibrils by different levels of methodology representing different rigor, accuracy, and efficiency. The binding sites and energies are also compared to CBD and AD tau by docking and conventional MD simulations. Most of these sites contain at least one polar/charged residue. This feature is present for all of the sites identified by the docking, MD, and metadynamics simulations in this study. The docking studies indicate that **CBD2115**, **PBB3**, and **PMPBB3** exhibit higher binding probabilities and affinities for many surface sites in PSP tau than other tracers, in contrast to the stronger binding for the concave sites on AD tau fibrils. Our MD simulations clearly showed that due to the differences between the folding patterns between PSP/CBD tau and AD tau, one and the same tracer behaves differently on the surface sites of these tau fibrils. For the identified unstable docking sites, tracers can occasionally move to other sites that are low-ranked in the docking. For example, the closure and enlargement for the small groove (K^290^[C^291^][G^292^]S^293^[K^294^]D^295^ on PSP) and the big groove of the AD protofibril, respectively. The results may stress the importance of the induced-fit mechanism where the tracer and fibril mutually and dynamically perturb the structures of the counterpart. This process may also affect the so-called *k*_on_ and *k*_off_ reactivity constants for the binding of tracers. From metadynamics simulations, the preferred surface binding sites on PSP tau were investigated for CBD2115, PI2620, Flortaucipir, PMPBB3, PBB3, and MK6240. PMPBB3 and PBB3 exhibited stronger binding to the Q^276^[I^277^]I^278^, Q^351^[S^352^]K^353^, and N^368^[K^369^]K^370^ sites than the other tracers.

Our results can be divided into general and specific outcomes: (1) general in the sense of showing that, indeed, *in silico* modeling can act as a valuable trendsetter and give structure-property relation predictors providing tracer–fibril interaction patterns beneficial for further chemical radio labeling and *in vitro*/*in vivo* development; (2) specific because we could rank potent 4R tau PET tracers both with respect to effectivity and selectivity. We believe that exploring the binding characteristics of the tracers to PSP, CBD, and AD tau protofibrils can be valuable to establish and understand the structure–activity relationships between the known tracers and tau fibrils, as well as help to design novel high-affinity and selectivity PET tracers targeting a specific tau fibril.

## Methods

### Preparation of the Protofibrils

All of the initial structures of AD, CBD, and PSP protofibrils were taken from the Cryo-EM structures stored in the Protein Data Bank (PDB) with the PDB codes 7NRV,^28^6VHA,^38^ and 7P65,^15^ respectively. For CBD tau, we chose 6VHA because of its smaller buried cavity compared to 6TJX (Figure S6).^16^ For AD tau, 7NRV was selected because it was fitted with the existence of **PMPBB3**.^28^ For PSP tau, 7P65 is selected because it is from the canonical tissue with Richardson’s syndrome.^15^ Considering the movement along the principal axis of fibrils and the consumption of computational resources, a nine-chain protofibril was constructed for each Cryo-EM structure of PSP, CBD, and AD using the method mentioned in our previous study.^35,49^ The automated preparation protocol implemented in the Protein Preparation Workflow of the Schrödinger Suite (Version 2021-4) was used,^50,51^ which assigns the atom types, bond orders, hydrogen atoms, and protonation states of ionizable residues at the pH of 7.4 and minimizes the system by the convergence of heavy atoms to 0.3 Å of root-mean-square deviations (RMSD) with the OPLS4 force field.^52^ The assigned protonation states of the ionizable residues for each prepared protofibrils were visually inspected.^53^

### Molecular Docking

The structures of the selected tracer molecules were either downloaded from PubChem (https://pubchem.ncbi.nlm.nih.gov/) or manually depicted in the Maestro interface of the Schrödinger Suite, followed by the preparation with the LigPrep module for the assignment of atom types, bond orders, and atomic partial charges.^54^ All of the docking runs were accomplished by the Glide module in the Schrödinger Suite,^55,56^ with the same protocol as in our previous study.^35^

### Molecular Dynamics Simulations

For the tracer binding sites identified by docking, molecular dynamics simulations were carried out using the Desmond package (version 2021-4) with an OPLS4 force field.^52,57^ A box with an orthorhombic shape was used to place each initial protofibril–tracer complex with a minimum buffering area of 10 Å to the boundary of the filled TIP3P water molecules.^58^ Sodium or/and chloride ions were added to neutralize the system and to increase the concentration of salt to 0.15 M. The default energy minimization schemes were applied, followed by the relaxations using the Nose–Hoover thermostat and Martyna–Bobias–Klein barostat for 10.0 and 20.0 ps, respectively.^59,60^ After that, a 100-ns production simulation was performed in the NPT ensemble (300 K and 1 atm) for each system. The trajectories were inspected by Visual Molecular Dynamics (VMD, version 1.9.4a57) software with the in-house Tcl scripts to calculate the contacts.^61^ The accumulated percentage of contacts was calculated by counting the total number of protein–ligand atom pairs within 4 Å over all of the snapshots in the simulations for each ligand.

### Metadynamics Simulations

To thoroughly explore the binding and unbinding of the tracer on the surface sites, we further performed well-tempered metadynamics simulations for each tracer on PSP protofibrils with a randomly placed position.^62^ The simulation protocols were adopted as our previous studies^35,49^ using PLUMED (version 2.8.0) patched with GROMACS (version 2021.4).^63−65^ Briefly, the tracers were parameterized with the general Amber force field with the charges fitted by the restrained electrostatic potential procedure, in which the electrostatic charges were firstly calculated by Gaussian 16 (rev. C01) at the Hartree–Fock level using the 6–31G(d) basis set.^66^ The Amber 99SB-ildn force field and TIP3P model were applied for the protein and solvent atoms, respectively.^58,67,68^ A 4.5-μs production run was performed for each tracer in the NVT ensemble (*T* = 300 K), using the 2D (*x* and *y*) coordinates of the center of mass of the tracer as collective variables (CVs), with a ±5 Å restraint on the *z* coordinates (along the principal axis of the protofibril) of the tracer’s center of mass. To calculate the biased potential along CVs, the initial Gaussian height and the bias factor were set at 0.2 kcal/mol and 6 with an interval of 1000 steps, respectively.^69^ The free energy surface (FES) was obtained by reweighting the biased potential for the sampled CV space with 1000 bins.^45^

## Supporting Information Available

Inner cavities of selected tau structures; Autodock4 results; binding modes of tracers in site s6 after MD simulations; mobility of **PI2620** in the V2 site; most contact residue profile of PSP tau derived from 100- and 500-ns MD simulations; and Cryo-EM structures of CBD tau protofibrils (PDF)
