# Supplementary material for: Insight into the Binding of First- and Second-Generation PET Tracers to 4R and 3R/4R Tau Protofibrils
Source: ACS Chem Neurosci. 2023 Aug 28;14(18):3528–39. doi: 10.1021/acschemneuro.3c00437 (PMC10515481; doi:10.1021/acschemneuro.3c00437)
Supplement: Supplementary file 1 — cn3c00437_si_001.pdf [file cn3c00437_si_001.pdf]

Supporting Information

Insight into the Binding of First- and Second-Generation PET Tracers to 4R and 3R/4R tau Protofibrils

Junhao Li[a*], Amit Kumar[b], Bengt Långström[c], Agneta Nordberg[b,d], and Hans Ågren[a,e*]

[a] *Department of Physics and Astronomy, Uppsala University, Box 516, SE-751 20 Uppsala, Sweden*
[b] *Department of Neurobiology, Care Sciences and Society, Division of Clinical Geriatrics, Center for Alzheimer Research, Neo 141 84 Stockholm*
[c] *Department of Chemistry - BMC, Uppsala University, Box 516, SE-751 20 Uppsala, Sweden*
[d] *Theme Aging Karolinska University Hospital, S-141 86, Stockholm, Sweden*
[e] *College of Chemistry and Chemical Engineering, Henan University, Kaifeng, Henan 475004, P.R. China*

Correspondence: Junhao Li, junhao.li@physicus.uu.se;
Hans Ågren (hans.agren@physics.uu.se)

KEYWORDS: 4R tau fibrils, metadynamics, positron emission tomography tracer, free energy surface, Alzheimer disease, molecular dynamics

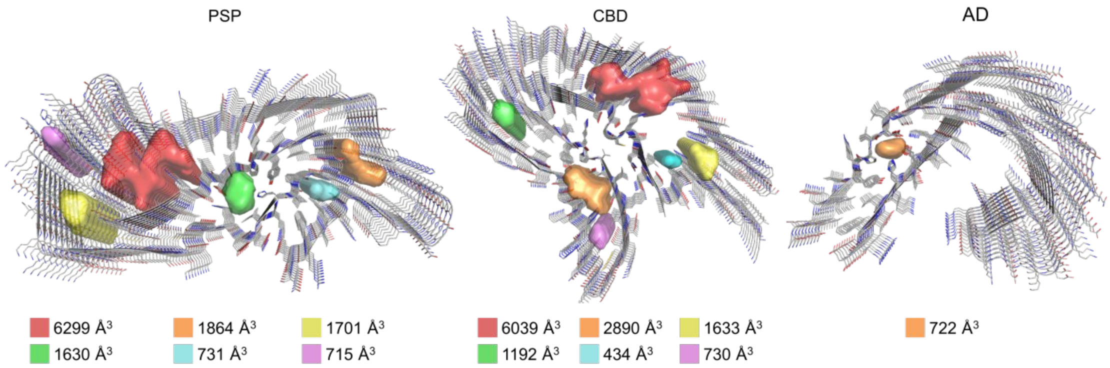

Figure S1. Volumes of the interior cavities in the 9-chain PSP,[1] CBD,[2] and AD[3] protofibrils identified by pyVOL (version 1.7.8).[4] The minimum and maximum probe radii are 1.4 and 4.5 Å, respectively. The partitioning parameters, max number of sub-pockets and sub-pocket radius, are set to 12 and 1.7 Å, respectively.

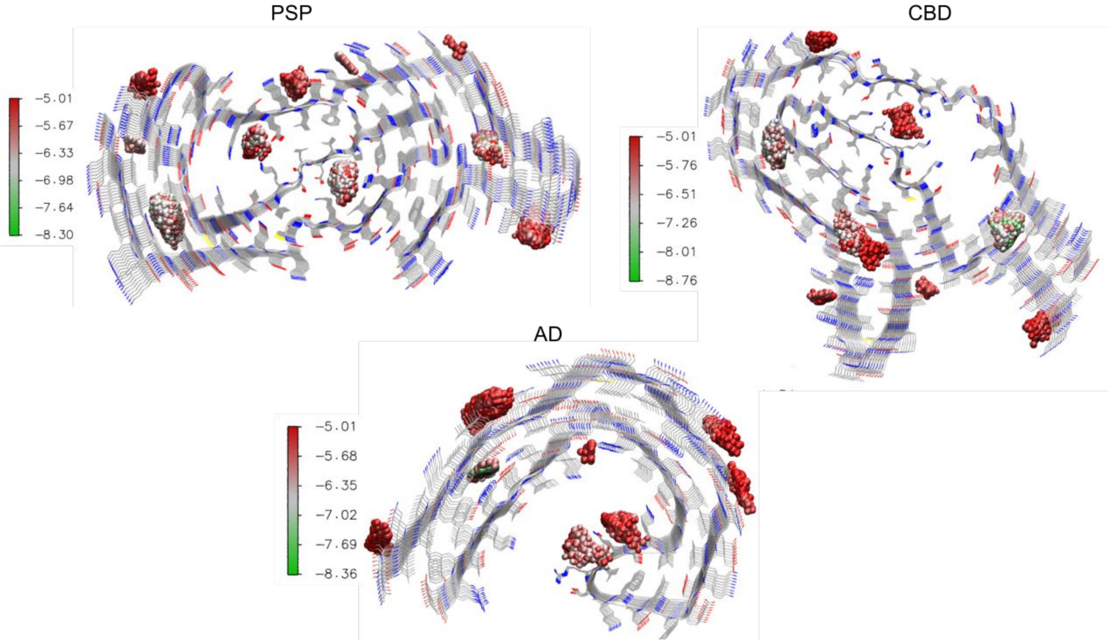

Figure S2. Scoring and binding sites of all the tracers docked to PSP, CBD and AD protofibrils by Autodock 4.[5] The setting of grid box is similar to previous study,[6] in which a very large box covering the entire protofibril with 2000 output poses for each tracer. All the poses with docking score lower than -5.0 kcal/mol were depicted as spheres with the color scaled by docking scores.

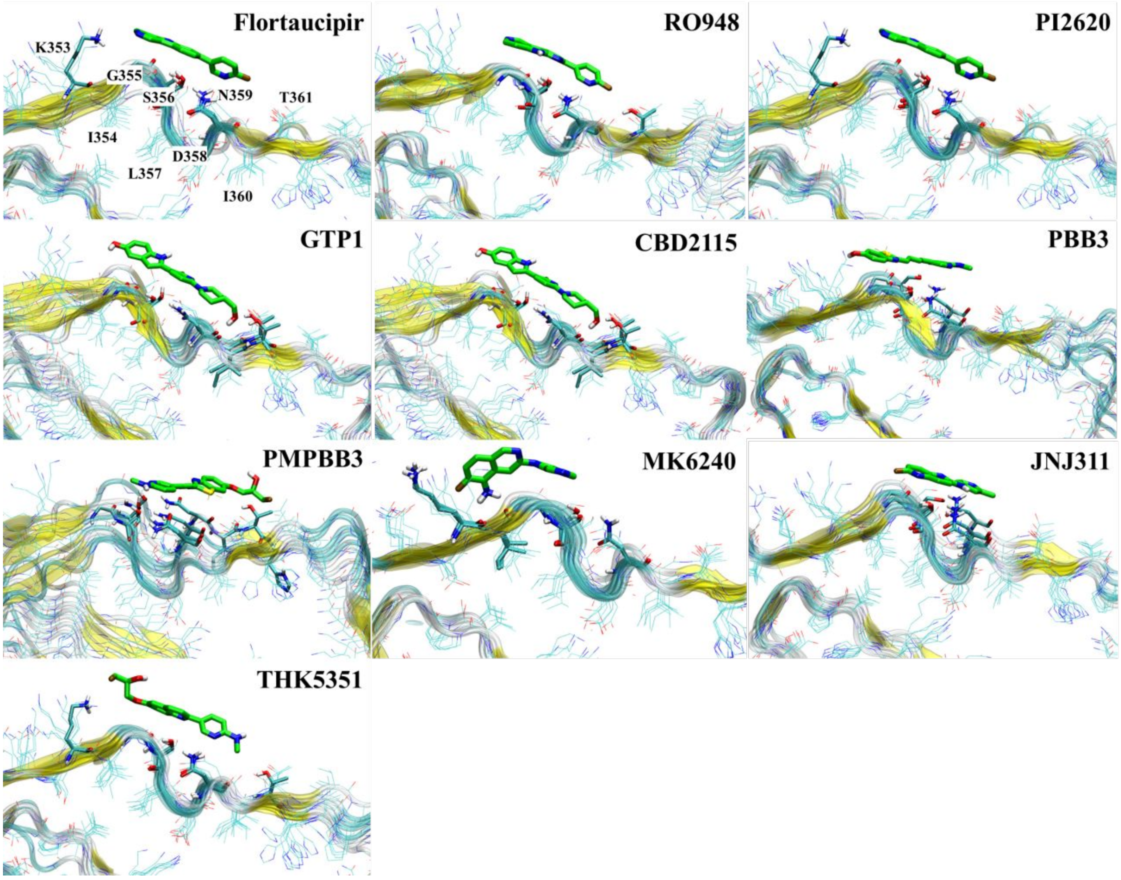

Figure S3. Tracer binding modes that is perpendicular to the axis of protofibril observed during the MD simulations starting from site s6 of the CBD protofibril.

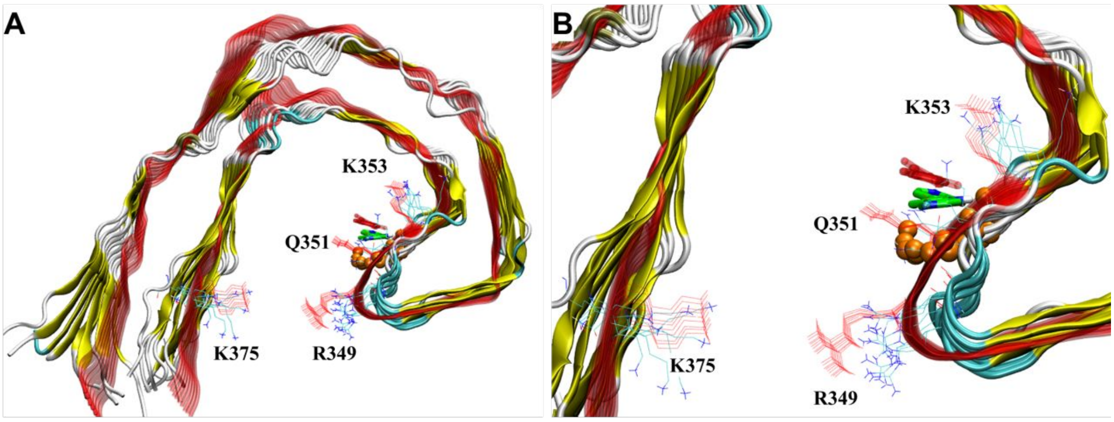

Figure S4. Positional shift of **PI2620** in the V2 site and the enlargement of the entire groove of the AD protofibril. The initial conformation, PI2620, and the contacted atoms (within 4 Å of tracer heavy atoms) are colored in red, green sticks, and orange spheres, respectively.

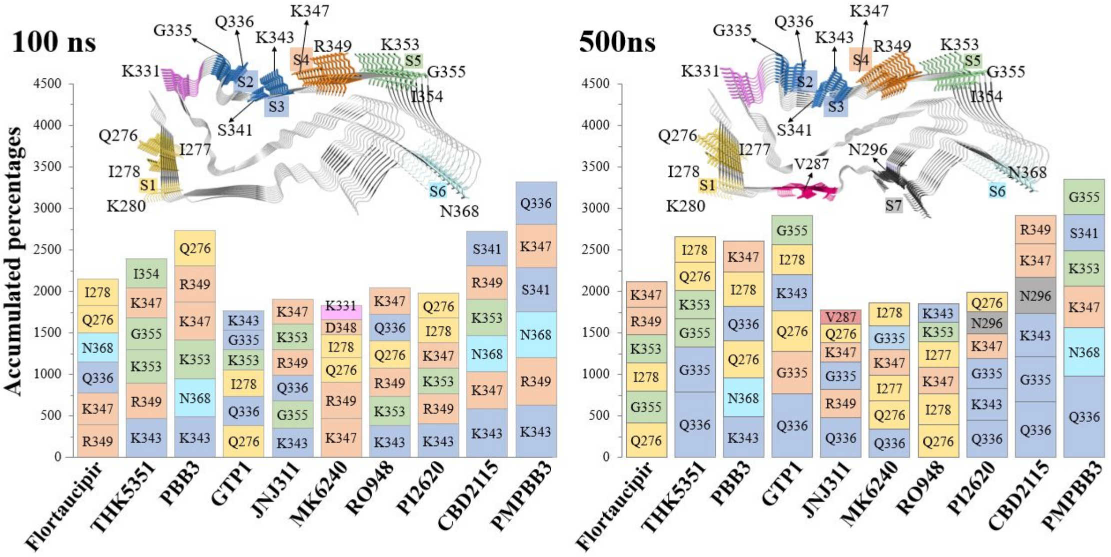

Figure S5. Top-6 most contacted residues for each tracer on PSP protofibril from 100-ns (left) and 500-ns (right) MD simulations.

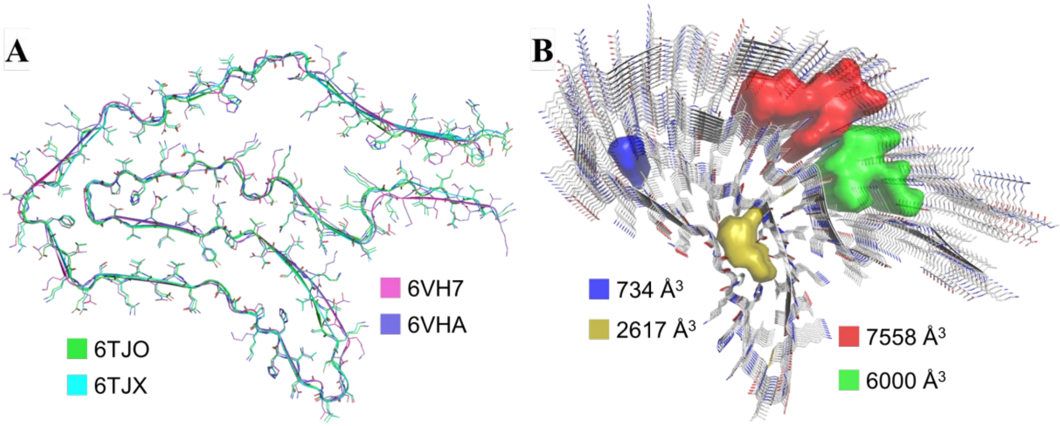

Figure S6. Comparison of the cryo-EM structures of CBD protofibril. A. Superimposition of the single-chain structures from 6TJX,[7] 6TJO,[7], 6VH7,[2] and 6VHA.[2] B. Distribution and volumes of the detectable internal cavities in 6TJX structure by pyVOL with the same setting of parameters used in Figure S1.

References

1. Shi, Y.; Zhang, W.; Yang, Y.; Murzin, A. G.; Falcon, B.; Kotecha, A.; van Beers, M.; Tarutani, A.; Kametani, F.; Garringer, H. J.; Vidal, R.; Hallinan, G. I.; Lashley, T.; Saito, Y.; Murayama, S.; Yoshida, M.; Tanaka, H.; Kakita, A.; Ikeuchi, T.; Robinson, A. C.; Mann, D. M. A.; Kovacs, G. G.; Revesz, T.; Ghetti, B.; Hasegawa, M.; Goedert, M.; Scheres, S. H. W., Structure-based Classification of Tauopathies. *Nature* **2021**, *598*, 359-363.
2. Arakhamia, T.; Lee, C. E.; Carlomagno, Y.; Duong, D. M.; Kundinger, S. R.; Wang, K.; Williams, D.; DeTure, M.; Dickson, D. W.; Cook, C. N.; Seyfried, N. T.; Petrucelli, L.; Fitzpatrick, A. W. P., Posttranslational Modifications Mediate the Structural Diversity of Tauopathy Strains. *Cell* **2020**, *180*, 633-644.e612.
3. Shi, Y.; Murzin, A. G.; Falcon, B.; Epstein, A.; Machin, J.; Tempest, P.; Newell, K. L.; Vidal, R.; Garringer, H. J.; Sahara, N.; Higuchi, M.; Ghetti, B.; Jang, M.-K.; Scheres, S. H. W.; Goedert, M., Cryo-EM Structures of tau Filaments from Alzheimer's Disease with PET Ligand APN-1607. *Acta Neuropathol.* **2021**, *141*, 697-708.
4. Ryan, H. B. S.; Arvin, C. D.; Avner, S., PyVOL: a PyMOL plugin for visualization, comparison, and volume calculation of drug-binding sites. *bioRxiv* **2019**, 816702.
5. Morris, G. M.; Huey, R.; Lindstrom, W.; Sanner, M. F.; Belew, R. K.; Goodsell, D. S.; Olson, A. J., AutoDock4 and AutoDockTools4: Automated Docking with Selective Receptor Flexibility. *J. Comput. Chem.* **2009**, *30*, 2785-2791.
6. Kuang, G.; Murugan, N. A.; Zhou, Y.; Nordberg, A.; Ågren, H., Computational Insight into the Binding Profile of the Second-Generation PET Tracer PI2620 with Tau Fibrils. *ACS Chem. Neurosci.* **2020**, *11*, 900-908.
7. Zhang, W.; Tarutani, A.; Newell, K. L.; Murzin, A. G.; Matsubara, T.; Falcon, B.; Vidal, R.; Garringer, H. J.; Shi, Y.; Ikeuchi, T.; Murayama, S.; Ghetti, B.; Hasegawa, M.; Goedert, M.; Scheres, S. H. W., Novel tau Filament Fold in Corticobasal Degeneration. *Nature* **2020**, *580*, 283-287.
